# The GuLF STUDY: A Prospective Study of Persons Involved in the *Deepwater Horizon* Oil Spill Response and Clean-Up

**DOI:** 10.1289/EHP715

**Published:** 2017-03-31

**Authors:** Richard K. Kwok, Lawrence S. Engel, Aubrey K. Miller, Aaron Blair, Matthew D. Curry, W. Braxton Jackson, Patricia A. Stewart, Mark R. Stenzel, Linda S. Birnbaum, Dale P. Sandler, for the GuLF STUDY Research Team

**Affiliations:** 1 Epidemiology Branch, National Institute of Environmental Health Sciences (NIEHS), National Institutes of Health (NIH), Department of Health and Human Services (DHHS), Research Triangle Park, North Carolina USA; 2 Department of Epidemiology, UNC Gillings School of Global Public Health, Chapel Hill, North Carolina, USA; 3 Office of the Director, NIEHS, NIH, DHHS, Research Triangle Park, North Carolina, USA; 4 Occupational and Environmental Epidemiology Branch, Division of Cancer Epidemiology and Genetics, National Cancer Institute, NIH, DHHS, Rockville, Maryland, USA; 5 Social & Scientific Systems, Inc., Durham, North Carolina, USA; 6 Stewart Exposure Assessments, LLC, Arlington, Virginia, USA; 7 Exposure Assessment Applications, LLC, Arlington, Virginia, USA

## Abstract

**Background:**

The 2010 *Deepwater Horizon* disaster led to the largest ever marine oil spill. Individuals who worked on the spill were exposed to toxicants and stressors that could lead to adverse effects.

**Objectives:**

The GuLF STUDY was designed to investigate relationships between oil spill exposures and multiple potential physical and mental health effects.

**Methods:**

Participants were recruited by telephone from lists of individuals who worked on the oil spill response and clean-up or received safety training. Enrollment interviews between 2011 and 2013 collected information about spill-related activities, demographics, lifestyle, and health. Exposure measurements taken during the oil spill were used with questionnaire responses to characterize oil exposures of participants. Participants from Gulf states completed a home visit in which biological and environmental samples, anthropometric and clinical measurements, and additional health and lifestyle information were collected. Participants are being followed for changes in health status.

**Results:**

Thirty-two thousand six hundred eight individuals enrolled in the cohort, and 11,193 completed a home visit. Most were young (56.2% ≤ 45 years of age), male (80.8%), lived in a Gulf state (82.3%), and worked at least 1 day on the oil spill (76.5%). Workers were involved in response (18.0%), support operations (17.5%), clean-up on water (17.4%) or land (14.6%), decontamination (14.3%), and administrative support (18.3%). Using an ordinal job exposure matrix, 45% had maximum daily total hydrocarbon exposure levels ≥ 1.0 ppm.

**Conclusions:**

The GuLF STUDY provides a unique opportunity to study potential adverse health effects from the *Deepwater Horizon* oil spill.

**Citation:**

Kwok RK, Engel LS, Miller AK, Blair A, Curry MD, Jackson WB II, Stewart PA, Stenzel MR, Birnbaum LS, Sandler DP for the GuLF STUDY Research Team. 2017. The GuLF STUDY: a prospective study of persons involved in the *Deepwater Horizon* oil spill response and clean-up. Environ Health Perspect 125:570–578; http://dx.doi.org/10.1289/EHP715

## Introduction

The *Deepwater Horizon* (*DWH*) drilling rig explosion in April 2010 resulted in the largest marine oil spill in U.S. history (National Commission on the BP Deepwater Horizon Oil Spill and Offshore Drilling 2011). An estimated 4.9 million barrels of oil was released into the Gulf of Mexico from the time the *DWH* exploded until the well was capped on 15 July 2010. Approximately 1,100 linear miles of visible oiling occurred from Texas to the Florida panhandle (Michel et al. 2013). Tens of thousands of individuals participated in oil spill response and clean-up (OSRC) activities, including drilling relief wells, burning oil, cleaning the waters, marshes, beaches, and shoreline structures, decontaminating vessels and other equipment, and providing support to operations in multiple locations on and off the water. These activities exposed workers to heat stress, environmental contaminants, and injury. Nearly all efforts were completed by 30 June 2011.

Worker exposures varied over time both in relation to capping the well and clean-up needs. As a result of weathering, the composition of the leaked oil changed over time. The dispersants COREXIT® 9500 and COREXIT® 9527 were applied to break down the released oil. Additionally, a large volume of oil was burned, generating potentially harmful air pollutants (National Commission on the BP Deepwater Horizon Oil Spill and Offshore Drilling 2011). OSRC workers were potentially exposed to chemicals associated with crude oil, dispersants, and oil combustion products, with exposure levels depending on their job/tasks, location, and dates of work (Funk et al. 2011).

The OSRC workforce included individuals from the Gulf states and across the United States and comprised oil industry workers, Coast Guard and other government personnel, temporarily out-of-work fishermen participating in the Vessels of Opportunity program, individuals looking for work, and volunteers. OSRC workers who were Gulf coast residents may have been doubly affected because they may have encountered the same chemical/physical exposures in coastal residences as the OSRC workers experienced in their jobs (Savitz and Engel 2010). Additionally, major industries in the region were disrupted, resulting in job loss and reduced income for many residents in affected communities, possibly increasing emotional distress, domestic violence, and substance abuse (Aguilera et al. 2010; Laffon et al. 2016).

Potential health consequences of the crude oil, dispersant, and particulate exposures include respiratory, neurological, hepatic, renal, endocrine, hematological, and other systemic effects (Aguilera et al. 2010; Laffon et al. 2016). Of the 38 major reported oil spills before the *DWH* disaster, only 7 were studied for human health effects. Most studies were cross-sectional and investigated acute health symptoms. In many studies, exposure status was based on residential address in relation to the oil spill location or on performance of a small number of clean-up tasks. Studies with prospective data were generally small and had short follow-up. A number of the studies reported respiratory symptoms, including cough and shortness of breath, among exposed persons (Laffon et al. 2016). In a follow-up study 1–2 years after exposure, clean-up workers (Zock et al. 2007) had persistent though reduced excess risk of lower respiratory tract symptoms with evidence of increasing risk with increasing degree of exposure. Others (Meo et al. 2009) reported reduced forced vital capacity, forced expiratory volume in 1 sec, forced expiratory flow, and maximum voluntary ventilation among other clean-up workers. Other commonly reported acute symptoms include itchy eyes, nausea and vomiting, dizziness, headaches, and dermatological problems (Laffon et al. 2016). Given the limited information on the long-term health effects of oil spills and the magnitude of the *DWH* disaster, the Director of the National Institutes of Health, Dr. Francis Collins, charged the National Institute of Environmental Health Sciences (NIEHS) to examine the potential human health effects of the disaster. This paper describes the study design, characteristics of the study cohort, and plans for follow-up.

## Methods

The GuLF STUDY (Gulf Long-term Follow-up Study) is a prospective cohort study designed to examine human health effects among the *DWH* OSRC workers. It targeted these workers because they were likely to have the greatest potential for direct physical contact with the crude oil, dispersants, and oil combustion products. Outcomes of interest were derived from the literature on health effects of oil spills, studies of petroleum-exposed workers, NIOSH (National Institute for Occupational Safety and Health) surveillance reports during the spill, and media and community reports of symptoms among oil spill workers and residents of nearby communities.

The study protocol was reviewed by the Institute of Medicine in September 2010 (Institute of Medicine 2010) and was approved by the Institutional Review Board of the NIEHS. The study is overseen by a Scientific Advisory Board and a Community Advisory Board.

### Recruitment and Eligibility

We assembled a master recruitment list from training and badge records, BP (the Responsible Party for the spill) contractors, a NIOSH Roster, and local, state, and federal workers (Appendix 1). Most individuals were required to have completed safety training and to scan an ID badge each time they accessed any controlled areas. However, the quality of the information on these lists varied, with many key pieces of personal information (e.g., first name, phone number, Social Security number) missing or misspelled/misentered. There was also a substantial amount of duplicate records. Extensive data cleaning and tracing efforts were needed to construct a final master list.

Individuals with contact information were considered eligible for the study if they were ≥ 21 years of age at enrollment and had either worked on the OSRC in any capacity for at least 1 day or had completed safety training but were not hired. Enrollment occurred between March 2011 and May 2013. Potential participants were mailed an invitation, brochure, and privacy statement and given 2 weeks to opt out before telephone interviewers attempted contact. Interviewers called each number at least 12 times. The calling cycle was repeated after an interval of inactivity in order to reach seasonal workers and others away from their residence for short periods. Call attempts were also repeated after contact information was updated using a commercial tracing service. Postcards were mailed to eligible participants to encourage them to call the study toll-free number to enroll.

Broad-based recruitment activities ended 31 December 2012, but efforts continued through May 2013 to increase enrollment of particular groups, including Vietnamese-speaking participants and those with the greatest exposure potential (e.g., workers at the source of the spill).

### Community Outreach

A comprehensive outreach plan promoted participation across the region. Before launch, the NIEHS hosted public meetings and webinars to solicit input from key stakeholders. An intensive media campaign included advertisements in newspapers, television, radio, billboards, social media, and electronic bulletin board outlets, endorsements from the Surgeon General and regional and national celebrities. Study investigators were interviewed on television and radio and in print media to promote enrollment. Targeted groups included potential study participants, families of workers, community leaders, and others who could legitimize the study and encourage enrollment.

To reach potential Vietnamese-speaking participants, we enlisted the assistance of trusted community partners from groups serving local Vietnamese communities. Oil and gas industry professionals were underrepresented on the master recruitment list, largely because they were already trained and were not required to complete the new safety training for OSRC work. To find such workers, we placed recruiters at the heliport serving oil and gas professionals in Houma, Louisiana, over a 12-week period, to distribute study recruitment materials and obtain contact information.

### Enrollment Interview

After providing verbal consent, participants completed a 30- to 60-min computer-assisted telephone enrollment interview (NIEHS 2011); the length depended on the extent and duration of a participants’ OSRC activities. In addition to information related to OSRC activities, participants provided demographic, socioeconomic, occupation, lifestyle, and health information, including symptoms experienced during the time of the oil spill and at the time of the interview. Where possible, the questionnaire used validated or previously used questions from major epidemiologic studies and national surveys to facilitate comparisons (Hamilton et al. 2011). Interviews were conducted in English and Spanish. An abbreviated version of the questionnaire was administered to participants who spoke only Vietnamese. The questionnaires can be found at https://www.niehs.nih.gov/gulfstudy.

### Home Visit

At the conclusion of the enrollment interview, English- and Spanish-speaking participants from eastern Texas, Louisiana, Mississippi, Alabama, and Florida were invited to participate in a home visit. Because visit scheduling required a separate phone call from the home examiner, some who initially agreed were lost. Several tracing efforts, including door-to-door canvassing, helped to locate participants and schedule visits.

The home visit included an additional interview, collection of biological and environmental samples, and anthropometric/physiologic measurements. Before the visit, participants received instructions regarding the visit, answers to frequently asked questions, a copy of the consent form, and a sterile urine collection cup with instructions for collecting a clean catch first morning void on the day of the home visit. Trained certified medical assistants carried out the visits using centrally provided equipment and supplies. Written informed consent was obtained. Additional information on OSRC work, physical and mental health, lifestyle, and occupational, residential and family health histories was obtained via computer-assisted interview. Participants received a $50 gift card for completing the home visit. To enhance enrollment, participants who completed their home visit were also eligible to be randomly selected to receive a $500 gift card. There were three drawings for every 5,000 participants, with a total of six gift cards given in different regions of the Gulf.

***Anthropometric and clinical measurements.*** Height, weight, hip and waist circumference, and resting blood pressure and heart rate were recorded using standardized protocols (Hamilton et al. 2011). Spirometry was performed according to American Thoracic Society/European Respiratory Society standards using a portable ultrasonic spirometer (Easy on-PC; ndd Medical Technologies). A spirometry expert reviewed all tests and scored the results independently.

***Biological and environmental sample collection.*** A total of 52.5 mL of venous blood was collected from each participant. A small subgroup provided additional blood samples for quality assurance. Saliva for DNA analysis (Oragene DNA; DNA Genotek) was collected if blood could not be collected. If the participant had not collected a first morning void, a clean catch spot urine sample was collected during the visit. A hair sample was collected if the participant’s hair was at least 1 cm long. Toenail clippings were collected from each toe. If possible toenail samples were too short, participants were given a self-collection kit to mail samples to us.

Study staff recorded GPS coordinates at the doorstep and collected alcohol dust wipe samples from the participant’s house. For a small subset of participants in selected counties/parishes in Alabama and Louisiana, a vacuum dust sample was also collected. Additional details about biological and environmental specimen collection, processing, handling, shipping, and storage are available elsewhere (Engel et al., in press).

***Participant reports and medical referral.*** At the conclusion of the home visit, participants were given reports with their body mass index, blood pressure, and dipstick urinary glucose test results and interpretation. Medical referrals were given if requested. After centralized review and interpretation, results from pulmonary function tests were mailed to participants with the previously shared findings and recommendations for seeking care. Abnormal results were sent to the participant’s physician if requested.

Field staff were trained to identify urgent physical or mental health issues (e.g., hypertensive crises or acute mental distress). If necessary, participants were referred to a nearby federally qualified health center or emergency facility. Field staff contacted emergency services when needed, and participants were connected to suicide prevention hotlines when appropriate.

### Exposure Assessment

OSRC workers performed a range of jobs/tasks, from stopping the leak to administrative support, with different exposure profiles (Table 1). Initially, jobs and tasks were the basis of a preliminary exposure assessment. Due to the weathering of the oil, vessel, vessel type, location, and time periods were later identified as possible determinants of individual exposure levels. The ultimate goal of the GuLF STUDY is to have quantitative exposure estimates for total hydrocarbons (THC) and BTEX-H (benzene, toluene, ethylbenzene, xylene, hexane) as these oil-related chemicals comprised most of the air measurements taken during the spill and are generally considered to be the more toxic components. Exposure estimates for dispersants and particulates from burning were also desired because of their association with some health effects and because of concerns raised by the public. An ordinal job–exposure matrix (JEM) was developed based on jobs or tasks/vessel or vessel type/location/time period to estimate THC exposures for study participants (Stewart et al., in press). THC is a composite of the volatile chemicals from the oil and, as such, can be thought of as a surrogate for the “OSRC oil experience.” In the development of the questionnaire and the ordinal and quantitative JEMs, study industrial hygienists (IHs) relied on BP measurement data and their accompanying documentation, federal and BP contractor reports, numerous other spill-related documents, and interviews with key personnel managing the OSRC effort and some workers.

**Table 1:** Types of jobs/tasks performed during oil spill response: GuLF STUDY 2011–2013.

Job class	Examples of typical jobs/tasks
Response	Jobs on rig vessels attempting to stop the oil release or drilling the relief well Jobs on vessels that could see the wellhead Environmental sampling on the water
Support of operations	Operational support:
	Refueling vehicles
	Moving hazardous materials (e.g., oily boom)
	Operating heavy equipment
Clean-up on water	Searching for or collecting oil from the water:
	On a vessel handling boom
	On a vessel skimming oil
	On a vessel burning oil
Decontamination	Decontaminating vessels, boom, tanks, structures Handling/cleaning wildlife
Clean-up on land work	Patrolling beaches and marshes Cleaning/removing oil from beaches, marshes, and other shoreline structures Repairing oily boom
Administrative support	Aerial crew Food service Security Onsite/offsite driver Office work

The exposure section of the enrollment interview was structured to capture detailed information about the participants’ OSRC activities and served as the link to the JEM. Participants provided the start/stop dates for any OSRC work and then for each OSRC job/task queried, start/stop dates, average number of days worked/week, average number of hours worked/day, use of personal protective equipment, and dermal contact with chemical agents. Participants also provided information on heat stress and other work-related exposures and on sleeping quarters.

More than 28,000 full-shift, personal air monitoring samples were collected on workers by BP contractors to characterize exposure to OSRC chemicals from April 2010 through June 2011. Because multiple chemicals were analyzed on each sample, 160,000 measurements were available on THC, BTEX-H, and other toxicants. A large proportion of these measurements was below the reported limits of detection when analyzed based on occupational exposure limits. When these monitoring data were recalibrated by one of the BP contractors and the study IHs to reflect the analytical methods’ limits of detection, it was possible to quantify levels below the initially reported LODs. The effort substantially decreased the amount of censored data; for example, THC censored data went from 80% to ~ 20%. The proportion of censored data for the other chemicals was still relatively high (~ 70%) but was substantially lower than the original 95% censoring. We evaluated strategies for dealing with censored data and developed methods to leverage the censored data on THC to develop estimates for other BTEX-H chemicals (Huynh et al. 2014, 2016; Quick et al. 2014).

Our team of experienced IHs used the recalculated air measurement data to identify factors associated with exposure levels to characterize exposures: jobs/tasks, vessel/vessel type, location, and time period. Unique combinations of these factors were identified that were expected to have similar distributions of THC exposure. The measurement data were used to determine average THC exposures for each job or task/vessel or vessel type/location/time period combination (*n* = 2,385 “exposure groups”), which was translated to ordinal values (1–7). The resulting JEM was linked to the OSRC work reported in the questionnaire to estimate THC exposures for each participant in the cohort. Different metrics can be developed for different exposure–response scenarios and assumptions. For example, we estimated the maximum exposure by identifying the maximum level across all estimates assigned to an individual to create a person-specific maximum exposure metric. Exposure averages (mean or median) within and across jobs/tasks or in specific time periods (e.g., before the well was capped) or locations also can be developed.

Specific questionnaire responses were also used to identify, based on tasks, vessels, locations, and dates, workers with likely exposure to dispersants (yes/no) and to particulates (low, medium, high) from burning of oil. Quantitative exposure estimates for inhaled THC and specific chemicals (e.g., BTEX-H) are being developed, as are semiquantitative estimates of dermal exposure, estimates for dispersants, and estimates for particulate matter from burning.

### Long-term Cohort Follow-up

Participants receive annual newsletters, holiday cards, and other mailings, including an annual reminder to update contact information either through the study website (https://www.gulfstudy.nih.gov) or by calling a toll-free number. In addition to providing information about the study, these mailings keep the address database up-to-date.

Study participants will be followed via telephone interview every 2–3 years; the first round took place from May 2013 through May 2016. Participants who completed the home visit and the first follow-up telephone interview, living within ~ 60 miles of Mobile, Alabama, or New Orleans, Louisiana, were invited to participate in a comprehensive clinical examination, including collection of additional biological samples and tests of pulmonary and neurobehavioral function. The cohort will be followed for mortality and cancer incidence and, if feasible, for other outcomes using electronic medical records.

## Results

### Full Cohort

Our primary sources of names for recruitment included a roster of workers compiled by NIOSH and clean-up training records provided by a BP contractor (PEC Safety, Mandeville, LA). After de-duplicating these source files, we identified 113,096 presumably unique individuals, but only 44,103 had sufficient contact information for recruitment. We supplemented our primary source files with 18,700 unique names from a variety of other sources (Appendix 1). Thus, our recruitment master file consisted of 62,803 apparently unique individuals with presumed accurate contact information. After placing calls to the names on file, we determined that 1,182 were duplicates, 308 were deceased, 1,135 were ineligible, and 1,255 had communication difficulties or were unavailable during the time window, leaving 58,923 presumably eligible participants. Of these, 22,572 opted out or broke off telephone contact before eligibility was determined. Of the remaining 36,351 individuals (62% of known eligible participants with usable contact information), 32,608 completed the enrollment telephone interview (90% of those confirmed eligible; 55% of potentially eligible participants). Of these, 999 participants completed an abbreviated interview in Vietnamese. Participants represent the full range of worker identification sources (Table 2).

**Table 2:** Source of contact information for persons enrolled in the GuLF STUDY 2011–2013.

Source list^*a,b*^	Number enrolled (32,608)	Proportion doing clean-up work (77%)
PEC training list	22,467	72
NIOSH roster	1,142	84
Vessels of opportunity (VOO)	267	89
U.S. Coast Guard	2,992	74
TRG badging data	3,417	94
U.S. and Florida Fish & Wildlife	671	97
Other federal agency^*c*^	720	86
Rig workers from POB and THR lists	139	95
Heliport recruitment	128	92
Other	665	86
Abbreviations: POB, persons on board; THR, time history report. ^***a***^Hierarchical listing in order shown to eliminate inclusion of workers on more than one list. ^***b***^See Appendix 1 for definitions. ^***c***^National Oceanic and Atmospheric Administration, Agency for Toxic Substances and Disease Registry, U.S. Geological Survey, Department of the Interior.		

**Figure 1:**
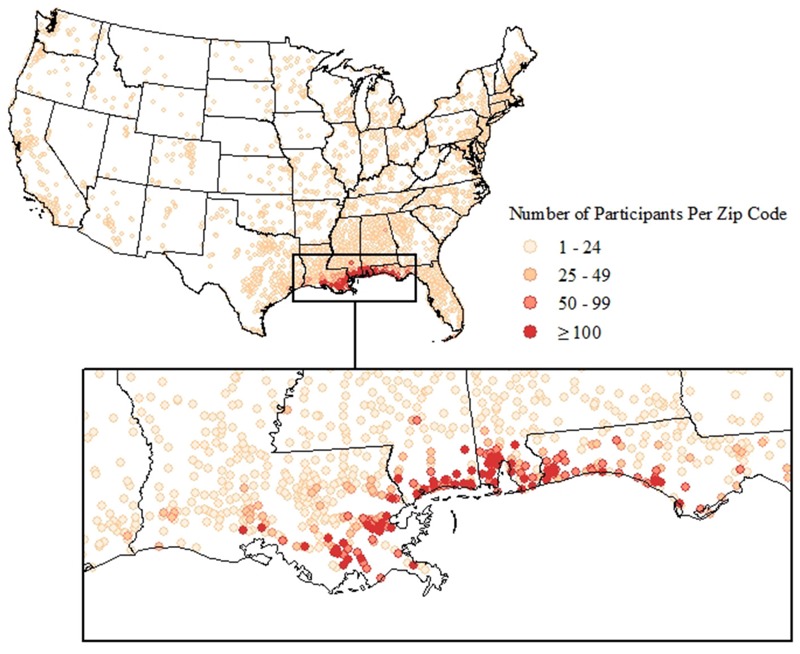
Residential location of GuLF STUDY participants across the United States, 2011–2013.

The majority (82.3%) lived in Alabama, Florida, Louisiana, Mississippi, or Texas (Table 3). The remainder, including responders from the Coast Guard and other federal agencies (e.g., the U.S. Fish and Wildlife Service, National Oceanic and Atmospheric Administration), as well as others with unique skills or interest in job opportunities, came from elsewhere in the United States (Figure 1). The majority were ≤ 45 years old (56.2%), male (80.8%), married (56.2%), and had an annual household income ≤ $50,000 (54.1%), with nearly 40% reporting their race as nonwhite (22.8% black, 4.1% Asian, 9.3% other/multi-racial).

**Table 3:** Characteristics at enrollment: full cohort, Gulf state residents, and home visit participants: GuLF STUDY 2011–2013 [*n* (%)].

Subject characteristics	Full cohort, total (*N *= 32,608)	Full cohort, workers (*N *= 24,937)	Full cohort, nonworkers (*N *= 7,671)	Home visit, eligible Gulf residents^*a*^ (*N *= 25,304)	Home visit, completed (*N *= 11,193)
Age (years)
< 30	6,262 (19.2)	5,014 (20.1)	1,248 (16.3)	4,915 (19.4)	1,973 (17.6)
30–45	12,074 (37.0)	9,532 (38.2)	2,542 (33.1)	9,122 (36.0)	3,931 (35.1)
> 45	14,160 (43.4)	10,308 (41.3)	3,852 (50.2)	11,190 (44.2)	5,282 (47.2)
Don’t know/refused	112 (0.3)	83 (0.3)	29 (0.4)	77 (0.3)	7 (0.1)
Sex
Male	26,341 (80.8)	20,578 (82.5)	5,763 (75.1)	20,360 (80.5)	8,752 (78.2)
Female	6,265 (19.2)	4,359 (17.5)	1,906 (24.8)	4,942 (19.5)	2,441 (21.8)
Don’t know/refused	2 (0.0)		2 (0.0)	2 (0.0)
Race
White	20,688 (63.4)	16,097 (64.6)	4,591 (59.8)	15,634 (61.8)	6,106 (54.6)
Black	7,425 (22.8)	5,626 (22.6)	1,799 (23.5)	6,943 (27.4)	3,881 (34.7)
Asian	1,325 (4.1)	781 (3.1)	544 (7.1)	218 (0.9)	76 (0.7)
Other/multi-racial	3,026 (9.3)	2,329 (9.3)	697 (9.1)	2,417 (9.6)	1,094 (9.8)
Don’t know/refused	144 (0.4)	104 (0.4)	40 (0.5)	92 (0.4)	36 (0.3)
Hispanic ethnicity
Yes	2,115 (6.5)	1,711 (6.9)	404 (5.3)	1,604 (6.3)	676 (6.0)
No	30,399 (93.2)	23,159 (92.9)	7,240 (94.4)	23,626 (93.4)	10,487 (93.7)
Don’t know/refused	94 (0.3)	67 (0.3)	27 (0.4)	74 (0.3)	30 (0.3)
Location at enrollment
Alabama	5,919 (18.2)	4,491 (18.0)	1,428 (18.6)	5,838 (23.1)	2,959 (26.4)
Florida	6,975 (21.4)	5,031 (20.2)	1,944 (25.3)	6,898 (27.3)	3,223 (28.8)
Louisiana	7,856 (24.1)	5,599 (22.5)	2,257 (29.4)	7,293 (28.8)	2,743 (24.5)
Mississippi	4,241 (13.0)	3,316 (13.3)	925 (12.1)	3,974 (15.7)	1,930 (17.2)
Texas	1,837 (5.6)	1,521 (6.1)	316 (4.1)	1,301 (5.1)	338 (3.0)
Other	5,780 (17.7)	4,979 (20.0)	801 (10.4)
Marital status
Married/living as married	18,337 (56.2)	14,096 (56.5)	4,241 (55.3)	13,531 (53.5)	5,577 (49.8)
Divorced/separated/widowed	6,137 (18.8)	4,593 (18.4)	1,544 (20.1)	5,223 (20.6)	2,610 (23.3)
Never married	7,840 (24.0)	6,066 (24.3)	1,774 (23.1)	6,418 (25.4)	2,961 (26.5)
Don’t know/refused	294 (0.9)	182 (0.7)	112 (1.5)	132 (0.5)	45 (0.4)
Educational attainment
Less than high school/equivalent	5,099 (15.6)	3,822 (15.3)	1,277 (16.6)	4,843 (19.1)	2,378 (21.2)
High school diploma/GED	9,436 (28.9)	7,158 (28.7)	2,278 (29.7)	8,319 (32.9)	3,789 (33.9)
Some college/2-year degree	9,382 (28.8)	7,301 (29.3)	2,081 (27.1)	7,552 (29.8)	3,351 (29.9)
4-year college graduate or more	7,584 (23.3)	6,026 (24.2)	1,558 (20.3)	4,504 (17.8)	1,640 (14.7)
Don’t know/refused	1,107 (3.4)	630 (2.5)	477 (6.2)	86 (0.3)	35 (0.3)
Annual household income
< $20,000	8,414 (25.8)	6,150 (24.7)	2,264 (29.5)	7,740 (30.6)	4,165 (37.2)
$20,001–$50,000	9,235 (28.3)	7,153 (28.7)	2,082 (27.1)	7,505 (29.7)	3,461 (30.9)
> $50,000	11,185 (34.3)	9,042 (36.3)	2,143 (27.9)	7,411 (29.3)	2,771 (24.8)
Don’t know/refused	3,774 (11.6)	2,592 (10.4)	1,182 (15.4)	2,648 (10.5)	796 (7.1)
Worked ≥ 1 day(s) on clean-up
Yes	24,937 (76.5)	24,937 (100.0)		18,943 (74.9)	8,968 (80.1)
No	7,671 (23.5)		7,671 (100.0)	6,361 (25.1)	2,225 (19.9)
^***a***^Gulf state residents eligible for home visit: Alabama, Florida, Louisiana, Mississippi, eastern Texas.					

Most participants worked ≥ 1 day(s) on clean-up (76.5%). There were few noteworthy differences between workers and those who trained but were not hired (nonworkers). Fewer workers than nonworkers were > 45 years of age (41.3% vs. 50.2%) and fewer were women (17.5% vs. 24.8%). More workers than nonworkers lived outside of the Gulf states (20.0% vs. 10.4%).

Most participants worked for a BP contractor (68.3%) or were affiliated with federal, local, or state government agencies (20.0%) (Table 4). Most reported multiple OSRC jobs/tasks (mean, 8.8 ± 8.5), and all but 13.5% worked before the well was capped. Only 2.6% were still working at study enrollment. We grouped workers hierarchically into broad job/task classes (Table 1), starting with the class having the greatest potential for THC exposure; 18% of workers ever worked jobs/tasks associated with the response (well capping) activities, 17.5% worked in jobs/tasks associated with support of operations, 17.4% conducted tasks associated with water clean-up, 14.3% had decontamination (e.g., vessels, equipment) jobs/tasks, 14.6% conducted tasks associated with clean-up on land, and 18.3% provided administrative support. A total of 9.4% of workers reported tasks and locations that were consistent with potential use of or exposure to dispersants, and 9.6% were consistent with potential exposure to particulate and other burning oil toxicants. Finally, 54.8% of workers were estimated to have a maximum exposure < 1.0 ppm, and only 13.8% had exposures ≥ 3.0 ppm.

**Table 4:** Exposure characteristics of oil spill response and clean-up workers—full cohort and home visit subcohort: GuLF STUDY 2011–2013 [*n* (%)].

Exposure characteristic	Full cohort (*N *= 24,937)	Home visit (*N *= 8,968)
Work affiliation
BP contractor	17,030 (68.3)	7,494 (83.6)
BP employee	622 (2.5)	232 (2.6)
Federal government	4,363 (17.5)	352 (3.9)
Local or state government	635 (2.6)	207 (2.3)
Volunteer	384 (1.5)	180 (2.0)
Other	1,029 (4.1)	385 (4.3)
Don’t know/refused	874 (3.5)	118 (1.3)
Number of jobs/tasks
1	4,965 (19.9)	913 (10.2)
2–5	6,295 (25.2)	1,719 (19.2)
6–10	5,863 (23.5)	2,428 (27.1)
≥ 11	7,814 (31.3)	3,908 (43.6)
Duration of work
≤ 14 days	1,463 (5.9)	445 (5.0)
15–180 days	18,122 (72.7)	6,278 (70.0)
> 180 days	5,352 (21.5)	2,245 (25.0)
Work timing^*a*^
Only before capping	4,194 (16.8)	1,338 (14.9)
Only after capping	3,355 (13.5)	1,043 (11.6)
Before and after capping	17,388 (69.7)	6,587 (73.5)
Still working at time of interview	650 (2.6)	246 (2.7)
Job class^*b*^
Response	4,479 (18.0)	1,680 (18.7)
Support of operations	4,371 (17.5)	1,888 (21.1)
Clean-up on water	4,328 (17.4)	1,319 (14.7)
Decontamination	3,561 (14.3)	1,794 (20.0)
Clean-up on land	3,634 (14.6)	1,462 (16.3)
Administrative support	4,564 (18.3)	825 (9.2)
Potentially exposed to dispersants^*c*^
Yes	2,355 (9.4)	1,156 (12.9)
No	21,138 (84.8)	7,417 (82.7)
Unknown	1,444 (5.8)	395 (4.4)
Potentially exposed to burning/flaring (all participants)
Yes	2,400 (9.6)	823 (9.2)
No	22,032 (88.4)	7,975 (88.9)
Unknown	505 (2.0)	170 (1.9)
Burning/flaring level (non-Vietnamese-speaking participants)^*c*^
None	21,734 (89.2)	7,975 (88.9)
Low	54 (0.2)	18 (0.2)
Medium	1,844 (7.6)	709 (7.9)
High	238 (1.0)	96 (1.1)
Unknown	505 (2.1)	170 (1.9)
Daily maximum THC ordinal level^*d*^
THC ≤ 0.29 ppm	5,458 (21.9)	1,264 (14.1)
THC 0.3–0.9 ppm	8,216 (32.9)	3,348 (37.3)
THC 1.0–2.99 ppm	7,791 (31.2)	3,014 (33.6)
THC ≥ 3 ppm	3,445 (13.8)	1,331 (14.8)
Unknown^*e*^	27 (0.1)	11 (0.1)
^***a***^Work relative to initial capping of well on 15 July 2010. ^***b***^Some people reported jobs or tasks in more than one job class. Assignments shown are hierarchical in the same order as listed. ^***c***^Not assessed for Vietnamese-only speaking participants (*n* = 562 workers). ^***d***^The daily maximum THC in parts per million across all jobs across all time periods. ^***e***^Exposure levels for those who began work after 30 June 2011 not estimated.		

### Home Visit Subcohort

**Figure 2:**
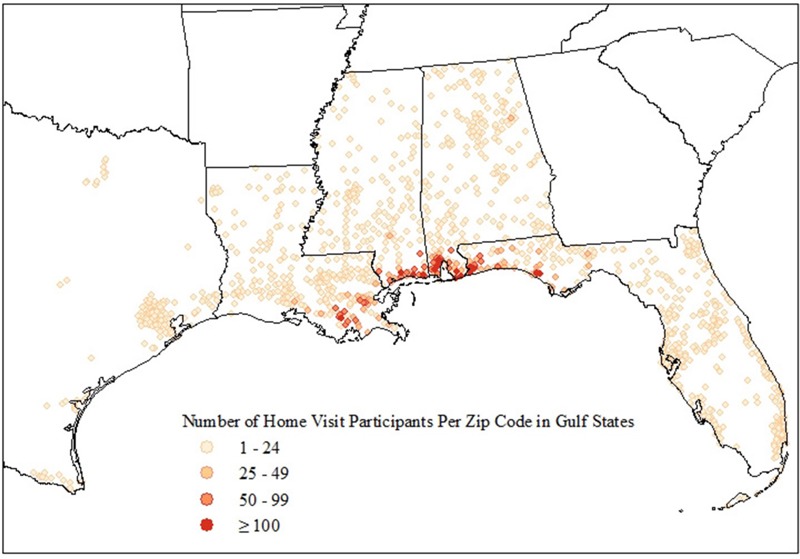
Residential location of GuLF STUDY home visit participants, 2011–2013.

A total of 25,304 English- or Spanish-speaking Gulf state residents were eligible for the home visit. Of those, 17,883 (70%) agreed to participate. However, 4,528 were lost to contact (25%) and 2,137 changed their minds (12%) before the home visit was scheduled. Of the 11,218 who completed a home visit (44% of those eligible and 63% of those who initially agreed), 25 had their home visits terminated early for health or safety reasons, leaving 11,193 with complete home visit exams. Most examination participants resided in the more highly affected counties/parishes along the coast of Louisiana, Mississippi, Alabama, and the Florida panhandle (Figure 2).

Characteristics of the Gulf state residents eligible for the home visit and those who completed a home visit are also shown in Table 3. Those who completed the home visit were older than those eligible (47.2% vs. 44.2% > 45 years of age). They were more often black (34.7% vs. 27.4%) and lower income (37.2% vs. 30.6% < $20,000). Home visit participants were more likely to have performed OSRC work (80.1% vs. 74.9%) and worked for a BP contractor, and to have reported more job/tasks, but were otherwise similar to the full cohort (Table 4).

## Discussion

The GuLF STUDY was created in response to public health concerns related to the largest marine oil spill in U.S. history. The study is investigating a wide range of potential physical and mental health outcomes among individuals engaged in cleaning up the *DWH* spill and is the largest study of its kind. It was designed as a prospective study to account for spatial and temporal variations in exposure as well as the large variety of OSRC jobs that participants performed. Studies of health effects of previous oil spills have generally had weaknesses that the GuLF STUDY addresses, including small sample size, cross-sectional designs focused on short-term outcomes, limited follow-up duration, or limited exposure assessment (Aguilera et al. 2010; Laffon et al. 2016). The GuLF STUDY also improves upon previous studies by using monitoring data collected at the time of the OSRC and extensive questionnaire data to estimate OSRC exposures and account for occupational history and potential confounders. The study is designed to evaluate both short- and long-term outcomes of interest with particular emphasis on respiratory and neurologic outcomes, which have been reported to manifest acutely with potentially persistent effects (Aguilera et al. 2010; Laffon et al. 2016). Although acute outcomes could not be captured in real time, we asked participants to report on symptoms they experienced at the time of the spill. This allows us to evaluate acute effects and, to the extent that such symptoms were or were not present at the time of interview, their persistence. We also hope to extend follow-up long enough to address community concerns about potentially increased cancer risk.

### Design Considerations

***Comparison groups.*** The choice of an appropriate comparison group is always difficult, but the selection was especially complex in this case. The *DWH* oil spill was unprecedented in size and scope. The majority of persons who worked on the OSRC were residents of the most highly affected counties/parishes along the Gulf. Thus, in addition to the potential for direct exposures to oil and dispersants during OSRC work, participants may have had OSRC-related exposures due to living near the coast that those living further away did not have. These include psychological and socioeconomic stressors associated with the closing of fisheries and reduced tourism and uncertainty about when the massive clean-up effort would be complete. By including predominantly local individuals who sought but did not obtain OSRC work, we included a comparison group who did not have work-related oil spill exposures, but who would potentially have similar nonoccupational oil spill experiences. Nonetheless, there were measured and potentially unmeasured differences between those who did and did not obtain OSRC work that may affect interpretation of health comparisons between these groups. We considered the possibility of including a comparison group from unaffected counties or states, but the Gulf Coast region differs substantially from others in major health indicators, industries, and sociodemographic factors.

GuLF STUDY participants encompass a range of OSRC experiences. This diversity of experiences will allow us to compare groups of workers who differ in their exposure to specific toxicants while taking into account other relevant measures associated with their nonoccupational experiences. Depending on the question of interest, workers can be compared with nonworkers or with workers who had lower levels of exposure to specific agents. Comparisons can be also restricted to subgroups defined by residence in or removed from affected communities.

***Participation rates.*** It is difficult to determine the exact number of OSRC workers. Our best estimate is ~ 110,000 to ~ 140,000 based on combining data from all of the sources used to develop the master recruitment list (Appendix 1). Even from those records, it was often difficult to tell whether we had unique names or duplicates, due to spelling errors and missing data fields. Contacting employers of the workers was not feasible because hundreds of contractors and subcontractors worked for BP. Despite our best efforts, we were able to obtain contact information for only 62,803 individuals. Much of the contact information that was collected from OSRC workers was intended for purposes other than research (e.g., for payroll). We lacked Social Security numbers for many workers, hindering some tracing efforts. Although this is not uncommon in the immediate demands of disaster response (Lurie et al. 2013), incomplete records with lack of secondary contact information to locate workers who moved or changed telephone numbers made contacting individuals difficult. Moreover, there was a tendency for multiple people to provide the same phone number or address (e.g., for a group home or trusted leader), and many provided only temporary information such as addresses of hotels, “flotels” (temporary living quarters for OSRC workers), or group homes where they lived only during the spill response.

We used a commercial tracing service to obtain the most recent contact information available on potential participants. This approach was most useful, however, for those with relatively complete personal information. The extent of discrepant information between the administratively collected contact information and that obtained through tracing highlights one of the challenges faced in locating disaster remediation personnel and members of highly mobile populations (Kennedy and Vargus 2001). In the GuLF STUDY, contact difficulties were exacerbated by the high use of disposable mobile phones and a tendency to inactivate and reactivate phone service. Once we were able to reach an individual, a number of factors could have contributed to nonparticipation, including distrust of the federal government and a litigious legal environment.

Although we cannot fully quantify the loss of contact, there is certainly potential for participation bias. Unfortunately, without any additional information about those who could not be reached or refused to participate, an accurate prediction about the magnitude and direction of any potential participation bias is impossible to make. Anecdotally, multiple factors were at work. Some lawyers who represented groups of workers requested that their clients join the study whereas others advised against it. Others could not be reached because they were gone for weeks or months at a time in pursuit of seasonal work, or their very early and long work hours made it difficult to participate. Those we could not reach could have been highly skilled technical workers no longer in the area or unskilled workers working in the underground economy. Thus without available data, it is impossible to know whether those who enrolled were healthier or less healthy than those who did not or whether participation is biased (e.g., whether exposed workers with health complaints were more likely to join). Although this could affect generalizability, comparisons within the cohort and among workers over time will be less affected. Furthermore, our analyses will benefit from being able to use both nonworkers and low-exposed workers as referent groups.

We collected data on many factors that could affect participation, such as being unemployed at the time of enrollment, worry about economic factors, and pre-spill health, and we will be able to take these factors into account when conducting within-cohort comparisons of those with the greatest and least degree of oil spill exposures. We do have limited demographic data from some lists of workers (e.g., the NIOSH roster), and comparisons of those who did and did not enroll in the study do not reveal obvious differences. We also have the ability to evaluate nonresponse bias by comparing those who were easy to recruit and those who required on-the-ground locating and multiple attempts to recruit and by comparing those who participated in the home visit and those who did not. Future analyses of exposure–outcome relationships will employ techniques such as inverse probability weighting to account for any informative losses.

***Exposure data.*** Previous studies of health effects associated with oil spills have relied on indirect measures such as distance from the spill or performance of a small number of clean-up tasks to characterize exposures (Laffon et al. 2016). Some have had biomonitoring data to classify exposures for small numbers of workers (Laffon et al. 2016). The GuLF STUDY is unique in the level of effort directed toward characterizing exposures. By taking advantage of and improving upon the > 28,000 personal air samples collected by BP contractors, we have been able to provide quantitative characterizations of chemical exposures due to OSRC work (Stewart et al., in press). We are also using other data such as information on days and locations of burning, weather conditions, and flight data for aircraft applying dispersants along with extensive questionnaire data to develop a range of qualitative, semiquantitative, and quantitative estimates to characterize exposures to oil and specific oil constituents, dispersants and particulate matter. Additional information on occupational history and occupational and nonoccupational exposures, including any oil industry–related exposures, was collected and will be considered in future analyses.

Our study was not funded until nearly 6 months after the disaster began. Although this was relatively soon after the disaster, we were unable to collect pre- and postexposure biological samples for exposure measurement. However, because the exposures varied so widely across jobs/tasks, location, and time, a single sample per individual would not have adequately captured the full range of exposures and could be used in only a limited way to validate questionnaire responses. Because there are no long-term biomarkers of relevant volatile compounds, the biological samples we did collect at enrollment will be of limited use for characterization of exposures during the height of OSRC activities.

In addition to exposures from OSRC chemicals, workers experienced a host of other stressors including physical (e.g., high heat and humidity, musculoskeletal strain, long working hours), financial (e.g., job loss), and psychological (e.g., depression, anxiety) stressors. The GuLF STUDY has attempted to capture a wide range of OSRC experiences and exposures to fully evaluate and understand the individual and combined effects of these stressors on health.

***Self-reported outcomes.*** Information on symptoms at the time of the spill was reported 1–3 years after the spill, leading to possible information loss and recall bias. Symptom reporting may also have been influenced by constant media attention to potential impacts of the spill. In an attempt to minimize reporting bias, the study interview did not anchor health-related questions in relation to the spill (e.g., we did not ask if symptoms had developed or worsened since the spill). Questions asked about current health and health at a specified time period in the past (not directly described as “before the spill”). Results related to health status at the time of enrollment or the home examination are also subject to bias if participation was related to health status or perceived exposures. Over time, the study will focus on specific diagnoses, some of which can be validated through medical records or other means.

***Collaborative opportunities.*** The prospective design of the GuLF STUDY allows for investigations of multiple health effects potentially associated with OSRC exposures and of new hypotheses that arise over time. The GuLF STUDY can serve as a resource for collaborative research with other intramural and extramural scientists interested in nested substudies and/or add-on studies of workers with specific exposures or outcomes of interest. Information on study resources and procedures for requesting access to study data or for proposing add-on studies can be found on the study website at https://www.gulfstudy.nih.gov.

## Conclusions

The GuLF STUDY is the largest oil spill–related study of its kind, with extensive data on both exposures and health outcomes related to OSRC work. The prospective design, collection of clinical data and biospecimens at baseline and at subsequent interviews/exams, and the development of quantitative estimates of OSRC exposures overcome many of the limitations of past studies, providing a unique platform for studies of potential health effects related to the diverse exposures associated with the spill. Because the population is racially and ethnically diverse and includes participants from communities that are understudied and medically underserved, it also represents an opportunity to address other important questions of public health concern.

## Appendix 1. Sources Used to Develop the Master Recruitment List. GuLF STUDY 2011–2013.

1. A partial voluntary roster of OSRC trainees developed by the National Institute for Occupational Safety and Health (NIOSH) (Funk et al. 2011)
2. Registration records of individuals who completed a safety training course conducted by PEC Safety (PEC), a BP contractor (http://pecsafety.com/)
3. Worksite security entrance/exit logs maintained (electronic badging) by The Response Group (TRG), a BP contractor (http://www.responsegroupinc.com/)
4. Employee identification lists from federal, state, and local agencies involved with the response effort such as the U.S. Coast Guard, U.S. Fish and Wildlife Service, National Oceanic and Atmospheric Administration (NOAA), Florida Fish & Wildlife Conservation Commission and others
5. Names and contact information collected during recruitment drives at the heliports where offshore oil and gas workers travel to and from offshore drilling rigs in the Gulf
6. Persons-on-Board (POB) lists from vessels involved with the response
7. Contact information from workers who wore personal monitoring badges as recorded on a Time History Report (THR)

## Appendix 2. GuLF STUDY Research Team

Sudipto Banerjee,^1^ Richard D. Cohn,^2^ Edward E. Gaunt,^2^ Caroline Groth,^3^ Audra B. Hodges,^2^ Tran Huynh,^4^ Robert L. Jensen,^5^ David A. Johndrow,^2^ John A. McGrath,^2^ Gurumurthy Ramachandran,^6^ Steven K. Ramsey,^2^ and Kathryn M. Rose^2^

^1^Department of Biostatistics, University of California, Los Angeles, Los Angeles, California, USA; ^2^Social & Scientific Systems, Inc., Durham, North Carolina, USA; ^3^Division of Biostatistics, University of Minnesota, Minneapolis, Minnesota, USA; ^4^Drexel University, Philadelphia, Pennsylvania, USA; ^5^Pulmonary Division, University of Utah Medical School, Salt Lake City, Utah, USA; ^6^Division of Environmental Health Sciences, University of Minnesota, Minneapolis, Minnesota, USA
